# Neuropsychiatric symptoms in limbic-predominant age-related TDP-43 encephalopathy and Alzheimer’s disease

**DOI:** 10.1093/brain/awaa315

**Published:** 2020-11-14

**Authors:** Kathy Y Liu, Suzanne Reeves, Kirsty E McAleese, Johannes Attems, Paul Francis, Alan Thomas, Robert Howard

**Affiliations:** 1 Division of Psychiatry, University College London, UK; 2 Translational and Clinical Research Institute, Newcastle University, Newcastle upon Tyne, UK; 3 Institute of Psychiatry, Psychology and Neuroscience, King’s College London, London, UK; 4 University of Exeter Medical School, Medical School Building, St Luke’s Campus, Exeter, UK

**Keywords:** Alzheimer’s disease, TDP-43, LATE, limbic, neuropsychiatric symptom

## Abstract

There is clinical overlap between presentations of dementia due to limbic-predominant age-related TDP-43 encephalopathy (LATE) and Alzheimer’s disease. It has been suggested that the combination of Alzheimer’s disease neuropathological change (ADNC) and LATE neuropathological changes (LATE-NC) is associated with greater neuropsychiatric symptom burden, compared to either pathology alone. Longitudinal Neuropsychiatric Inventory and psychotropic medication prescription data from neuropathologically diagnosed pure ADNC (*n = *78), pure LATE-NC (*n = *14) and mixed ADNC/LATE-NC (*n = *39) brain bank donors were analysed using analysis of variance and linear mixed effects regression models to examine the relationship between diagnostic group and neuropsychiatric symptom burden. Nearly all donors had dementia; three (two pure LATE-NC and one pure ADNC) donors had mild cognitive impairment and another two donors with LATE-NC did not have dementia. The mixed ADNC/LATE-NC group was older than the pure ADNC group, had a higher proportion of females compared to the pure ADNC and LATE-NC groups, and had more severe dementia versus the pure LATE-NC group. After adjustment for length of follow-up, cognitive and demographic factors, mixed ADNC/LATE-NC was associated with lower total Neuropsychiatric Inventory and agitation factor scores than pure ADNC, and lower frontal factor scores than pure LATE-NC. Our findings indicate that concomitant LATE pathology in Alzheimer’s disease is not associated with greater neuropsychiatric symptom burden. Future longitudinal studies are needed to further investigate whether mixed ADNC/LATE-NC may be protective against agitation and frontal symptoms in dementia caused by Alzheimer’s disease or LATE pathology.

## Teaser

LATE neuropathological change (LATE-NC) is associated with a dementia syndrome that clinically overlaps with Alzheimer’s disease. Liu *et al.* show that the combination of LATE-NC and Alzheimer’s disease is not associated with greater neuropsychiatric symptom burden than either pathology alone.

## Introduction

Limbic-predominant age-related TDP-43 encephalopathy (LATE) is a recently recognized disease entity, commonly affecting individuals over 80 years and diagnosed at autopsy (Nelson *et al.*, 2019). The defining neuropathological features of LATE are intracytoplasmic inclusions of phosphorylated nuclear protein TDP-43, collectively known as LATE-neuropathological change (LATE-NC), which stereotypically affects the amygdala, hippocampus and middle frontal gyrus in a hierarchical pattern, with or without coexisting hippocampal sclerosis pathology (Nelson *et al.*, 2019). LATE-NC and hippocampal sclerosis increase with advanced age and have been associated with an amnestic dementia syndrome that mimics Alzheimer’s disease dementia (Pao *et al.*, 2011; Brenowitz *et al.*, 2014). As TDP-43 deposition is also frequently associated with Alzheimer’s disease pathology, present in up to around 70% of Alzheimer’s disease cases (Josephs *et al.*, 2014, 2015), it has been proposed to play an important role in the clinical features associated with Alzheimer’s disease dementia.

Previous neuropathological studies have suggested that individuals with ‘pure’ LATE (where the sole presence of LATE-NC accounts for cognitive impairment), show slower clinical decline compared to those with ‘pure’ Alzheimer’s disease pathology, also known as Alzheimer’s disease neuropathological change (ADNC) [where the combined presence of hyperphosphorylated tau, amyloid-β protein and neuritic plaques account for cognitive impairment (Boyle *et al.*, 2017; Josephs *et al.*, 2017)]. In contrast, those with mixed ADNC and LATE-NC, i.e. both pathologies are present and fulfil specific pathological criteria, show greater cognitive impairment and faster decline compared to ‘pure’ LATE and ‘pure’ Alzheimer’s disease cases (Josephs *et al.*, 2014, 2015). However, this has not been consistently reported (Uryu *et al.*, 2008; Vatsavayi *et al.*, 2014). There is also evidence that LATE and Alzheimer’s disease may have distinct associated neurocognitive profiles; for example, higher verbal fluency and lower word list delayed recall test scores were seen in patients with hippocampal sclerosis pathology, 90% of whom were TDP-43 positive, versus those with Alzheimer’s disease pathology (Nelson *et al.*, 2011). As an important contributing factor to neurodegeneration with significant impact on public health, a greater understanding of LATE and its clinical features is needed.

There has been only limited research into the relationship between TDP-43 proteinopathy, predominantly located in limbic structures, and the nature and prevalence of neuropsychiatric symptoms in dementia. The limbic system consists of subcortical (including amygdala) and cortical (including hippocampus and orbitofrontal cortex) structures, and plays an important role in emotion, motivation and memory (Morgane *et al.*, 2005). In addition to LATE, other dementia pathological diagnoses, such as the behavioural variant of frontotemporal dementia, Parkinson’s, Huntington’s and Alzheimer’s diseases, can also involve the limbic system, which may underpin dementia-related neuropsychiatric symptoms, including psychosis, apathy, agitation and depression (Braak *et al.*, 1996; Shaw and Alvord, 1997; Rosenberg *et al.*, 2015; Miki *et al.*, 2016). These symptoms, which occur commonly in dementia and mild cognitive impairment (Lyketsos *et al.*, 2002), significantly reduce quality of life for patients (Wetzels *et al.*, 2010) and carers (Shin *et al.*, 2005), precipitate earlier institutionalization (Okura *et al.*, 2011) and are associated with more rapid disease progression and earlier death (Peters *et al.*, 2015). A study in individuals with Alzheimer’s disease found that the additional presence of LATE-NC was associated with an increased risk of agitation/aggression (Sennik *et al.*, 2017), while a further report found no association between TDP-43 pathology in the dentate gyrus and prefrontal cortex and psychosis in Alzheimer’s disease (Vatsavayi *et al.*, 2014). It is therefore still unclear whether LATE-NC, alone or in combination with Alzheimer’s disease pathology, contributes to neuropsychiatric symptoms, and if so, whether this can be accounted for by a greater degree of cognitive impairment.

The current study specifically investigated the relationship between LATE-NC and neuropsychiatric symptoms, by comparing clinical data between neuropathologically confirmed cases of pure LATE-NC, pure ADNC and mixed ADNC/LATE-NC. We tested the hypothesis that, after accounting for cognitive differences, the combined presence of LATE-NC and Alzheimer’s disease pathology would be associated with greater neuropsychiatric burden, compared to either pathology alone.

## Materials and methods

For this study, we used the Brains for Dementia Research (BDR) cohort of over 685 post-mortem human brains, donated between 2008 and 2018 to a network of six university brain banks (King’s College London, Bristol, Manchester, Oxford, Cardiff and Newcastle). Consent for clinical assessment, brain donation and storage, neuropathological assessment and data use for research was obtained in accordance with ethics approval 13/SC/0516 granted by the Oxford C Committee of the National Research Ethics Service. Further ethical, legal and recruitment details have previously been described (Francis *et al.*, 2018).

### Clinical and neuropathological assessment and diagnosis

During life, clinical assessments were conducted by a research nurse or psychologist. Baseline assessments were conducted face-to-face and follow-up assessments were conducted annually for participants with cognitive impairment, and between 1 and 5 years for cognitively healthy participants. A range of widely employed clinical assessment measures were used, including the Clinical Dementia Rating (CDR) global score, which was used to generate cognitive status (dementia, mild cognitive impairment or control) (see Francis *et al.*, 2018 for details). Following death, the right hemisphere, brainstem, and cerebellum were immersion fixed in 10% buffered aqueous formaldehyde solution for 4 weeks, followed by coronal dissection and paraffin-embedding. Standardized neuropathological assessment was performed for all cases and included the National Institute on Aging-Alzheimer’s Association (NIA-AA) criteria (Montine *et al.*, 2012). These were Thal phase assessment of amyloid-β deposition (Thal *et al.*, 2002), Braak staging of neurofibrillary tangle pathology (Braak *et al.*, 2006), Consortium to Establish a Registry for Alzheimer’s Disease (CERAD) protocol scoring for neuritic plaques (Mirra *et al.*, 1991), McKeith Lewy body stage (McKeith *et al.*, 2005) and cerebrovascular pathology contribution to cognitive impairment [vascular impairment neuropathological guidelines (VCING) (Skrobot *et al.*, 2016)]. Regarding LATE-NC, the amygdala and some or all of the following anatomical regions were examined for the presence of TDP-43 inclusions; subiculum, entorhinal cortex, hippocampus, middle temporal cortex, basal ganglia and middle frontal cortex. As these assessments predated the publication of recent consensus staging criteria for LATE-NC (Nelson *et al.*, 2019), only the presence or absence of LATE-NC and not the exact location was recorded, so the stage of LATE-NC could not be determined for the study. Neuropathological diagnoses were determined according to the type and anatomical distribution and fulfilment of appropriate pathological criteria of the most prevalent neuropathological lesion(s); if no other additional pathologies were present, the diagnosis was classified as ‘pure’, and if more than one criterion for the respective diagnoses was met, the diagnosis was classified as ‘mixed’.

### Study cohort selection

The MRC BBN database was searched for cases fulfilling neuropathological criteria for pure ADNC, pure LATE-NC and mixed ADNC/LATE-NC (Table 1). Of note, the criteria were inclusive of McKeith score of 0 and VCING score ‘low’, to ensure minimal confounding influence of Lewy body and vascular pathology. In total, data from 148 donors was selected, inclusive of 84 ‘pure’ ADNC, 22 cases of ‘pure’ LATE-NC and 42 cases of mixed ADNC/LATE-NC.

**Table 1:** Neuropathological diagnostic criteria for the three diagnostic groups

Diagnostic group	Neuropathological diagnostic criteria
Pure ADNC	Braak NFT stage V–VI Thal Phase 4–5 CERAD for neuritic plaques B or C McKeith score 0 VCING Low LATE–NC absent
Pure LATE-NC	Braak NFT stage 0–IV Thal Phase 0–5 CERAD for neuritic plaques 0–C McKeith score 0 VCING Low LATE–NC present
Mixed ADNC/LATE-NC	Braak NFT stage V–VI Thal Phase 4–5 CERAD for neuritic plaques B or C McKeith score 0 VCING Low LATE–NC present

### Neuropsychiatric symptoms

For inclusion in the analysis, donors had to have had at least one recorded neuropsychiatric assessment using the Neuropsychiatric Inventory (NPI) during life. The NPI provides a reliable and valid informant-based assessment of dementia-related behavioural symptoms (Cummings, 1997). It examines the following subdomains of behavioural functioning over the past month: delusions, hallucinations, agitation/aggression, dysphoria, anxiety, euphoria, apathy, disinhibition, irritability/lability, aberrant motor activity, night-time behavioural disturbances and appetite/eating abnormalities (Supplementary Table 1). If a caregiver indicates that the patient for whom they care experiences any of the behaviour subdomains, they are asked to rate the frequency on a 4-point scale, the severity on a 3-point scale and the distress the symptom causes on a 5-point scale. The domain total score is the product of the frequency score multiplied by the severity score for that behavioural domain, and the total NPI score is the sum total of all the individual domain scores (the carer distress level is not part of the total NPI score). As 15 donors did not have recorded NPI scores (four pure ADNC, eight pure LATE-NC and three mixed ADNC/LATE-NC donors), and two pure ADNC donors had retrospective assessments dated later than 1 month after the date of death, the final ‘NPI sample’ was *n = *131 (78 pure ADNC, 14 pure LATE-NC and 39 mixed ADNC/LATE-NC donors).

The primary measures of neuropsychiatric symptom burden used for analysis were total NPI score and four validated NPI factor subscale scores (Trzepacz *et al.*, 2013; van der Linde *et al.*, 2014), which have been proposed to represent major neuropsychiatric symptom clusters within patient subgroups. These are: ‘agitation’ (NPI-rated agitation/aggression, disinhibition, irritability and aberrant motor behaviour); ‘mood’ (NPI-rated depression, anxiety and irritability); ‘frontal’ (NPI-rated elation, apathy, disinhibition and irritability); and ‘psychosis’ (NPI-rated hallucinations and delusions).

A secondary measure of neuropsychiatric symptom burden was the number of prescribed psychotropic medications (commonly prescribed antidepressants, antipsychotics, benzodiazepines and z-drugs) recorded at each visit (Supplementary Table 2). We did not include anti-dementia medications (e.g. acetylcholinesterase inhibitors or memantine) as we believed they were less likely to be initiated in response to neuropsychiatric symptoms.

### Statistical analysis

The characteristics of the NPI sample were described using means and standard deviations (SD) or frequencies and proportions, as appropriate (Table 2). Missing data-points (Table 2) were omitted from cross-sectional analyses. Group means were compared using one-way ANOVA and proportions using chi-squared goodness-of-fit analyses. Cross-sectional analyses were performed in R version 3.5.1 and mixed effects regression analyses were performed using STATA/MP 16.0. The relationships between variables were tested at a significance level of α = 0.05.

**Table 2:** Characteristics of the three diagnostic groups from the NPI sample (*n* *=* 111)

	Diagnostic group		
	Pure ADNC (*n = *78)	Pure LATE-NC (*n = *14)	Mixed ADNC/LATE-NC (*n = *39)
Age at death, years^*^	80.3 [9.7]	88.1 [8.6]	86.2 [7.8]
Number of females (%)^*^	28 (35.9)	4 (28.6)	24 (61.5)
Years of education	12.8 [3.4] (M = 13)	11.7 [3.6] (M = 5)	11.9 [3.2] (M = 10)
Total number of assessments	2.6 [1.8]	2.6 [0.9]	2.8 [1.8]
Total follow up duration, months	31.7 [24.8]	29.0 [12.8]	30.8 [27.8]
Time between last assessment and death, months	10.4 [13.4]	6.8 [3.7]	7.6 [9.5]
Final MMSE at last assessment	15.3 [9.2] (M = 46)	17.0 [7.8] (M = 4)	10.0 [7.8] (M = 32)
Rate of MMSE decline, MMSE/year	−2.0 [2.1] (M = 60)	−1.6 [4.4] (M = 6)	−4.1 [3.3] (M = 33)
Final Global CDR at last assessment	2.4 [0.9] (M = 3)	1.9 [1.2] (M = 1)	2.8 [0.6] (M = 4)
Rate of Global CDR increase, CDR/year	0.2 [0.4] (M = 33)	0.1 [0.2] (M = 4)	0.3 [0.3] (M = 19)
Final CDR-SB at last assessment	11.4 [5.2]	8.8 [6.1]	12.5 [4.7] (M = 1)
Rate of CDR-SB increase, CDR/year	1.4 [1.9] (M = 29)	0.5 [3.3] (M = 2)	2.2 [3.5] (M = 13)
Cognitive status, *n* (%)	Dementia 75 (97.4), MCI 2 (2.5) (M = 1)	Dementia 10 (77.0), MCI 1 (7.7), no dementia 2 (15.4) (M = 1)	Dementia 38 (100) (M = 1)
Post-mortem delay, h	58.9 [34.2] (M = 1)	65.1 [25.9] (M = 2)	56.5 [39.6] (M = 1)
Total number of psychotropic medications	0.5 [0.9]	0.2 [0.4]	0.5 [0.6]

Values shown are mean (SD) unless % or rating indicated. If there were missing values, the number of missing values is indicated by (M). Cognitive status was based on the most recent global CDR score (dementia ≥1, mild cognitive impairment = 0.5, control = 0). CDR-SB = CDR Sum of Boxes; MCI = mild cognitive impairment.

* Categories with significant differences between the groups.

We initially performed cross-sectional analyses using one-way ANOVA to compare the mean and maximum total and factor NPI scores, and number of prescribed psychotropic medications, between the three neuropathological groups.

Linear mixed effects regression models were used to examine the longitudinal relationship between diagnostic group and measures of neuropsychiatric burden, over the follow-up period. The presence and strength of correlations in the pure LATE-NC and pure ADNC groups were compared to the mixed ADNC/LATE-NC (reference) group. Mixed effects models can account for the correlation between repeated measures due to unobserved inter-individual heterogeneity by incorporating random effects. They can also account for unequal follow up intervals by including time as a continuous variable, and for missing data by using maximum likelihood estimation, which uses all available data. We used random intercept models and tested the fit of adding random slopes to the model using the likelihood ratio test.

Regressions for each model were conducted before and after inclusion of cognitive [Mini-Mental State Examination (MMSE) score] and demographic factors (age, years of education and sex) as covariates. Donor age and MMSE scores recorded in the final assessment before death were used in the cross-sectional analyses and all repeated measures were included in the mixed models. As donors had different follow up intervals, time of follow up in months relative to death was also included as a covariate in the mixed models.

### Data availability

All clinical and neuropathological data is available via the Medical Research Council (MRC) brain banks network (BBN) database (https://brainbanknetwork.cse.bris.ac.uk). Data that support the analyses and findings in this study are available from the corresponding author upon reasonable request.

## Results

### Sample characteristics

The characteristics of the NPI sample are shown in Table 2. On average, participants were followed up for 30 months and had three assessments prior to death (Table 2 and Supplementary Table 3). Apart from differences in the ratio of males to females [χ^2^(2) = 14.3, *P *<* *0.001], age at death [*F*(2,128) = 8.1, *P *<* *0.001] and final global Clinical Dementia Rating (CDR) score [*F*(2,120) = 5.1, *P *=* *0.008], no other significant differences were found between the three groups. *Post hoc* tests showed that there were more females in the mixed ADNC/LATE-NC group compared to the pure ADNC (*P = *0.008) and pure LATE-NC groups (*P = *0.0340) (chi-squared test), a significantly older age of death in pure LATE-NC (*P = *0.009) and mixed ADNC/LATE-NC (*P = *0.004) than ADNC (Tukey’s test), and a higher final global CDR score in mixed ADNC/LATE-NC versus pure LATE-NC (*P = *0.007) (Tukey’s test). Based on the most recent CDR global score, nearly all donors had dementia; three (two pure LATE-NC and one pure ADNC) donors had mild cognitive impairment and another two donors with LATE-NC did not have dementia. On average, donors were rated as having moderate-severe dementia prior to death (mean final CDR scores were between 1.9 and 2.8). There were missing MMSE data from the final assessment for 82 individuals, which affected 59% of the pure ADNC group, 29% of the pure LATE-NC group and 82% of the mixed ADNC/LATE-NC group.

Regarding the degree of ADNC, all pure ADNC and mixed ADNC/LATE-NC samples were rated as ‘High’ and pure LATE-NC donors’ brains were rated as ‘Not’, ‘Low’ or ‘Intermediate’, according to the National Institute on Aging-Alzheimer’s Association guidelines (Hyman *et al.*, 2012). Additional neuropathological and cognitive status data for each LATE-NC donor are reported in Supplementary Table 4.

### Neuropsychiatric symptom burden between the three groups

There was no significant cross-sectional relationship between membership of a diagnostic group and mean or maximum total or factor NPI scores, or number of prescribed psychotropic medications. This remained the case after adjustment for cognitive and demographic variables.

Using linear mixed effects regression models (Table 3), membership of the pure ADNC group was associated with higher mean total NPI (11.5 points) and agitation factor (6.3 points) scores over follow-up, after, but not before, model adjustment for length of follow-up, cognitive and demographic factors, when compared to the mixed ADNC/LATE-NC group. The pure LATE-NC group also showed higher frontal factor subscores (4.5 points) after, and not before, model adjustment for these variables. No longitudinal relationship was observed between the diagnostic groups and mood and psychosis factor subscores, or number of prescribed psychotropic drugs. In the adjusted models, MMSE showed an inverse relationship to NPI total score and subscores for agitation, psychosis and frontal factor. Higher psychosis factor subscores were also associated with males compared to females. All longitudinal analyses were performed using random intercept models as the addition of random slopes did not improve model fit.

**Table 3:** Results from mixed effects regression models assessing the longitudinal relationship between measures of neuropsychiatric burden and diagnostic group

NPI score	Unadjusted model β (95% CI)		Adjusted model β (95% CI)						
	Group (versus mixed)		Group (versus mixed)		Sex (M versus F)	Age, years	Years of education	MMSE	Time, months
	Pure ADNC	Pure LATE-NC	Pure ADNC	Pure LATE-NC					
Total	4.9 (−2.6 to 12.3)	−1.1 (−13.0 to 10.8)	**11.5 (0.5 to 22.6)** ^*^	9.5 (−4.5 to 23.4)	3.2 (−5.1 to 11.6)	0.3 (−0.2 to 0.8)	−1.0 (−2.0 to 0.1)	**−0.9 (−1.3 to −0.5)** ^*^	0.0 (−0.1 to 0.2)
Agitation	2.0 (−1.4 to 5.3)	0.1 (−5.3 to 5.4)	**6.3 (1.6 to 10.9)** ^*^	4.3 (−1.5 to 10.2)	−0.0 (−3.5 to 3.5)	0.2 (−0.0 to 0.4)	−0.4 (−0.9 to 0.1)	**−0.4 (−0.6 to −0.2)** ^*^	0.0 (−0.0 to 0.1)
Mood	1.5 (−0.6 to 3.5)	1.2 (−2.1 to 4.4)	3.1 (−1.3 to 7.5)	1.2 (−4.3 to 6.8)	0.6 (−2.7 to 3.9)	0.1 (−0.1 to 0.3)	−0.4 (−0.8 to 0.1)	−0.1 (−0.2 to 0.1)	0.0 (−0.0 to 0.1)
Psychosis	0.6 (−0.8 to 2.1)	−0.2 (−2.5 to 2.1)	0.6 (−2.7 to 3.8)	0.1 (−4.0 to 4.2)	**2.8 (0.3 to 5.2)** ^*^	0.1 (−0.1 to 0.2)	−0.3 (−0.6 to 0.1)	**−0.2 (−0.3 to −0.0)** ^*^	−0.0 (−0.0 to 0.0)
Frontal	2.0 (−0.7 to 4.7)	0.4 (−4.0 to 4.7)	3.3 (−0.0 to 6.6)	**4.5 (0.3 to 8.8)** ^*^	0.2 (−2.3 to 2.7)	0.1 (−0.1 to 0.2)	−0.2 (−0.5 to 0.2)	**−0.3 (−0.5 to −0.2)** ^*^	0.0 (−0.0 to 0.1)
Total number of psychotropic drugs	0.0 (−0.3 to 0.3)	−0.3 (−0.8 to 0.1)	−0.2 (−0.5 to 0.2)	−0.4 (−0.9 to 0.1)	0.1 (−0.2 to 0.4)	−0.0 (−0.0 to 0.0)	−0.0 (−0.1 to 0.0)	−0.0 (−0.0 to 0.0)	−0.0 (−0.0 to 0.0)

Values are β-coefficients (to one decimal place) for total NPI score, four NPI factor subscales and total number of prescribed psychotropic drugs, measured in the NPI sample (*n *=* *131) using random effects models, with 95% confidence intervals (CI) before and after adjustment for cognitive (MMSE) and demographic (age, sex and years of education) factors, and follow up time relative to death (months).

The composite subscales were ‘agitation’ (NPI-rated agitation/aggression, disinhibition, irritability and aberrant motor behaviour); ‘mood’ (NPI-rated depression, anxiety and irritability); ‘frontal’ (NPI-rated elation, apathy, disinhibition and irritability); and ‘psychosis’ (NPI-rated hallucinations and delusions). F = females; M = males.

* Statistically significant results (*P *<* *0.05) are highlighted in bold, otherwise results were not significant.

## Discussion

We investigated whether the additional presence of LATE-NC in Alzheimer’s disease increased neuropsychiatric symptom burden, by comparing NPI data between pathologically confirmed mixed ADNC/LATE-NC versus pure ADNC and LATE-NC cohorts. Contrary to our hypothesis, we found that after adjustment for length of follow-up, cognitive and other demographic factors, mixed ADNC/LATE-NC was longitudinally associated with lower total NPI and agitation factor scores compared to pure ADNC, and lower frontal scores compared to pure LATE pathology.

A possible explanation for the appearance of significant differences in the adjusted, but not unadjusted models, was the presence of negative confounding (or potentially protective) factors on the relationship between diagnostic group and total agitation and frontal factor NPI scores. The mixed ADNC/LATE-NC group was significantly older than the pure ADNC group, in line with previous studies (Josephs *et al.*, 2008; Vatsavayi *et al.*, 2014) and had a higher proportion of females compared to the pure ADNC and LATE-NC groups (Table 2). Age-related LATE-NC is reported to occur in the oldest-old (Nelson *et al.*, 2019), and female sex has been associated with increased verbal agitation (Cohen-Mansfield and Libin, 2005) in Alzheimer’s disease. Both pure ADNC and LATE-NC groups showed higher correlation coefficients for total NPI, agitation and frontal factor scores in the adjusted versus unadjusted models, which supports the presence of negative confounding for these outcomes, and the lack of statistical significance in total NPI and agitation factor scores for the pure LATE-NC group might have been related to insufficient power due to the small sample size (*n = *14).

There was overlap between the frontal and agitation factor subscales, as both included the irritability and disinhibition/lability NPI subscales. Indeed, a *post hoc* analysis of individual NPI items (Supplementary Table 5), showed that the main findings appear to be driven by the agitation and disinhibition NPI items in the ADNC and pure LATE-NC groups respectively. Moreover, there was also neurobiological overlap, as agitation and frontal symptoms have been proposed to arise within a ‘dysexecutive syndrome’ due to fronto-subcortical circuit dysfunction (Lyketsos *et al.*, 2004). One possible explanation for our findings could be that the additional neuropathology associated with mixed ADNC/LATE-NC disrupts these neural circuits via neurodegenerative processes, resulting in attenuation of frontal and agitation symptom generation. Alternatively, it has been proposed that LATE-NC might protect against neuropsychiatric symptoms in Alzheimer’s disease (Vatsavayi *et al.*, 2014). A neuroprotective role of TDP-43 pathology, which originates in the amygdala and spreads to other limbic structures including frontal cortex, could also potentially explain the finding of lower agitation factor and total NPI scores in the mixed ADNC/LATE-NC versus pure ADNC group. A novel proposal, based on findings from this study, would be that mixed ADNC/LATE pathology, via a hitherto unknown mechanism, might be neuroprotective against agitation and frontal symptoms compared to either pathology alone. Although protein aggregates of hyperphosphorylated tau, amyloid-β and TDP-43 have been proposed to have additive pathogenic effects on neurodegeneration (Spires-Jones *et al.*, 2017), an alternative hypothesis is that these protein aggregates could represent protective processes against neuronal damage (Lee *et al.*, 2005; Bolognesi *et al.*, 2019).

Our findings contrast with those of a previous study that found an increased risk of NPI-rated agitation/aggression in individuals with mixed ADNC/LATE-NC compared with pure Alzheimer’s disease pathology (Sennik *et al.*, 2017). However, study findings may not be directly comparable due to differences in design. For example, a significant proportion of subjects in the earlier study had additional vascular and alpha-synuclein pathology, and multiple univariate analyses were performed (which the authors acknowledged may have inflated the chances of a type I error) with no covariate analysis to identify potential confounding factors, as years of education, MMSE and age differed between agitation positive and negative individuals.

We did not find a relationship between diagnostic group and the mood or psychosis factor scores, supporting findings from another study that found no effect of LATE-NC on psychosis in Alzheimer’s disease (Vatsavayi *et al.*, 2014). We and others (Vatsavayi *et al.*, 2014) found no association between diagnostic group and degree of cognitive impairment (MMSE score), in contrast to larger studies that have shown greater MMSE-related cognitive impairment associated with the presence of LATE-NC in Alzheimer’s disease (Josephs *et al.*, 2014). However, we did find a significantly higher final global CDR score in mixed ADNC/LATE-NC versus pure LATE-NC. In the mixed effects models, there was a small but significant effect of dementia severity (measured by lower MMSE) on neuropsychiatric symptom burden (NPI total score and subscores for agitation, psychosis and frontal factor subscores) across the diagnostic groups. This was consistent with earlier studies (Lyketsos *et al.*, 2000; Steinberg *et al.*, 2006). We also found higher psychosis factor subscores in males, although the literature is inconsistent, with the majority of studies in Alzheimer’s disease reporting no relationship between participant sex and presence of psychosis (Ropacki and Jeste, 2005; Steinberg *et al.*, 2006).

Our study had a relatively small number of donors with pure LATE-NC (*n = *14), which reflects the relatively rare prevalence of pure LATE-NC versus mixed pathology (James *et al.*, 2016). Of note, the majority (77%) of pure LATE-NC donors had dementia (based on CDR global score), who mostly, but not exclusively appeared to have higher Thal, Braak and CERAD scores versus those with MCI/no dementia (Supplementary Table 4 and Supplementary Fig. 1). However, due to the limited sample size, statistical within-group differences were not calculated, and this observation should be viewed cautiously. Regarding neuropathological assessment, LATE-NC was assessed as a dichotomous variable as LATE-NC staging data were not available. Again, because of the limited sample size of LATE-NC donors, additional staging information in this study would be interesting and any within-group differences would be at most, cautiously interpreted, but unlikely to change our main findings and conclusion. As LATE donors accumulate, staging of the sections from the 14 LATE-NC donors (precluded by the COVID-19 pandemic at the time of publication) and all future LATE-NC donors as per consensus criteria (Nelson *et al.*, 2019) should be completed to allow an adequately-powered within-group analysis in future. Although it is possible that data from a larger cohort of LATE-NC subjects might have increased the statistical power of between-group analyses, we still obtained positive findings from the comparison between pure ADNC and mixed ADNC/LATE-NC groups, and in the pure LATE-NC group. Our sample contained missing values, especially MMSE data, and although mixed effects models can account for this, there is still a possibility that this would have led to inadequate statistical power and influenced our findings. Although the NPI and its subscale factors have been shown to be valid outcome measures, they may not have been as sensitive or specific as others designed to measure a particular neuropsychiatric symptom in dementia. For example, the agitation factor was composed of NPI-rated agitation, irritability, disinhibition and motor disturbance, which may not have fully captured the complexity of the agitation construct compared to the Cohen-Mansfield Agitation Inventory (Finkel *et al.*, 1992) or the Agitated Behaviors in Dementia (ABID) scale (Logsdon *et al.*, 1999). Nonetheless, an advantage of using the NPI was the ability to investigate multiple neuropsychiatric symptoms in a single scale. Although we included the number of psychotropic drugs prescribed, we did not account for quantification of different doses or frequency of administration. A *post hoc* analysis showed that there were no significant relationships between individual drug classes (Supplementary Table 2) and diagnostic groups. We did not have data on concurrent physical health biomarkers at each assessment, such as routine blood tests, so were unable to account for the possible effect of acute physical illness. However, participants who were unwell may not have been able to complete all assessments. We also did not have data on the date of clinical dementia diagnosis or onset. Despite these limitations, a major strength of this study was the selection and comparison of pure cases of LATE-NC and ADNC without the presence of confounding (e.g. Lewy body or vascular) pathologies, using data from the BDR program with longitudinal standardized clinical assessments and highly characterized neuropathological data.

In conclusion, our findings do not support the hypothesis that LATE-NC in limbic regions in Alzheimer’s disease is associated with greater neuropsychiatric symptom burden. In contrast, our findings are more consistent with a possible protective effect of mixed ADNC/LATE-NC pathology on the risk of agitation in Alzheimer’s disease and of frontal symptoms in LATE. Future longitudinal studies in larger sample sizes are needed to replicate our findings and further define the clinical implications of LATE-NC in dementia.

## Supplementary Material

awaa315_Supplementary_Data

## Abbreviations

ADNC =: Alzheimer’s disease neuropathological change
CDR =: Clinical Dementia Rating
LATE-NC =: limbic-predominant age-related TDP-43 encephalopathy neuropathological change
MMSE =: Mini-Mental State Examination
NPI =: Neuropsychiatric Inventory
