# Supplementary material for: Neuropsychiatric symptoms in limbic-predominant age-related TDP-43 encephalopathy and Alzheimer’s disease
Source: Brain. 2020 Nov 14;143(12):3842–9. doi: 10.1093/brain/awaa315 (PMC7805786; doi:10.1093/brain/awaa315)
Supplement: awaa315_Supplementary_Data [file awaa315_supplementary_data.pdf]

SUPPLEMENTARY MATERIAL

Supplementary Table 1: Description of the Neuropsychiatric Inventory (NPI) subscales.

NPI subscale	Description
Delusions	Does the patient have false beliefs, such as thinking that others are stealing from him/her or planning to harm him/her in some way?
Hallucinations	Does the patient have hallucinations such as false visions or voices? Does he or she seem to hear or see things that are not present?
Agitation/Aggression	Is the patient resistive to help from others at times, or hard to handle?
Depression/Dysphoria	Does the patient seem sad or say that he /she is depressed?
Anxiety	Does the patient become upset when separated from you? Does he/she have any other signs of nervousness such as shortness of breath, sighing, being unable to relax, or feeling excessively tense?
Elation/euphoria	Does the patient appear to feel too good or act excessively happy?
Apathy/indifference	Does the patient seem less interested in his/her usual activities or in the activities and plans of others?
Disinhibition	Does the patient seem to act impulsively, for example, talking to strangers as if he/she knows them, or saying things that may hurt people's feelings?
Irritability/lability	Is the patient impatient and cranky? Does he/she have difficulty coping with delays or waiting for planned activities?
Motor disturbance	Does the patient engage in repetitive activities such as pacing around the house, handling buttons, wrapping string, or doing other things repeatedly?
Nighttime behaviors	Does the patient awaken you during the night, rise too early in the morning, or take excessive naps during the day?
Appetite/eating	Has the patient lost or gained weight, or had a change in the type of food he/she likes?

Supplementary Table 2: Drugs included in each psychotropic medication group. Lower case, upper case and title case capitalization for each word were included in the search.

Psychotropic medication group	Drug names included in search
Antidepressants	citalopram, trazodone, “triptyline”, venlafaxine, dosulepin, “oxetine”, phenelzine, sertraline, mirtazapine
Antipsychotics	olanzapine, risperidone, aripiprazole, quetiapine, haloperidol, amisulpride, sulpiride
Benzodiazepines	“iazepam”, lorazepam, midazolam, “emazepam”, alprazolam, “lonazepam”, “itrazepam”
Z-drugs	zopiclone, zolpidem, zaleplon

Supplementary Table 3: Follow up assessment frequency for the NPI sample (N=131)

Number of assessments	1	2	3	4	5	6	7	8
Number of participants	44	27	25	17	9	0	8	1

Supplementary Table 4: Detailed neuropathological data and cognitive status of LATE-NC donors from the BDR cohort. Participants 1-14 were included in the analysis and participants 15-21 (bold, italic font) were excluded from the analysis due to missing NPI data. Participant 14 had missing neuropathological and cognitive status data (NA). Neg= negative, MCI = mild cognitive impairment.

Participant	Thal phase	Braak stage	CERAD score	Cognitive status
1	5	4	B	Dementia
2	5	4	B	Dementia
3	3	4	B	Dementia
4	5	4	C	Dementia
5	4	4	B	Dementia
6	5	4	C	Dementia
7	5	4	B	Dementia
8	4	4	B	Dementia
9	4	1	A	Dementia
10	3	4	B	Dementia
11	2	3	neg	MCI
12	4	4	B	No dementia
13	4	4	B	No dementia
14	NA	NA	NA	NA
15	***2***	***1***	***A***	***Dementia***
16	***3***	***4***	***B***	***Dementia***
17	***1***	***2***	***neg***	***MCI***
18	***1***	***1***	***neg***	***No dementia***
19	***0***	***3***	***neg***	***No dementia***
20	***0***	***2***	***neg***	***No dementia***
21	***1***	***4***	***A***	***No dementia***
22	***0***	***2***	***neg***	***No dementia***

Supplementary Figure 1: 3D scatterplot showing Thal phase (x-axis), CERAD score (y-axis), Braak stage (z-axis) and cognitive status of 21 LATE-NC donors. CERAD score 0, 1, 2, 3 is equivalent to CERAD score negative, A, B, C in Supplemental Table 1 respectively.

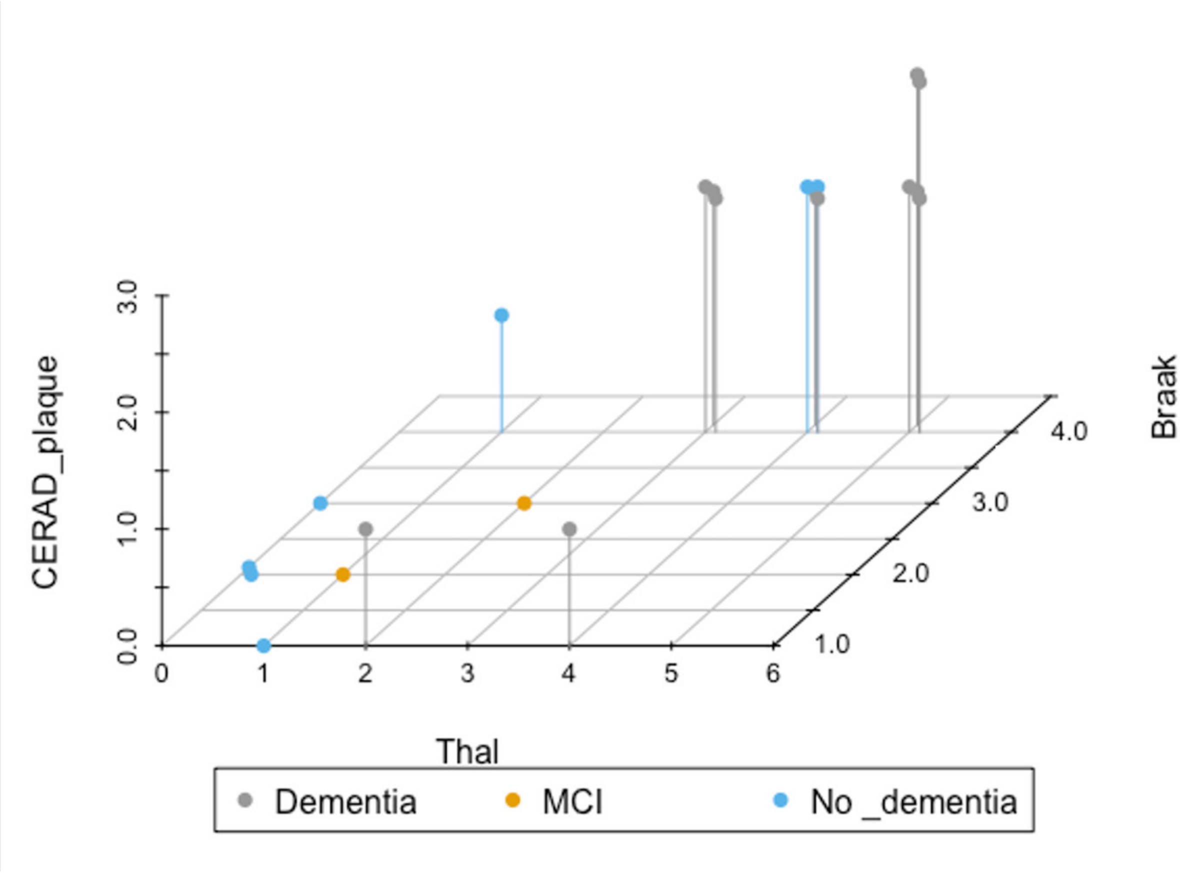

Supplementary Table 5: Results from mixed effects regression models assessing the longitudinal relationship between individual NPI items and diagnostic group. Values are β-coefficients (to one decimal place) for NPI subscales in the NPI sample (N=131) using random effects models, with 95% confidence intervals (CI) before and after adjustment for cognitive (MMSE) and demographic (age, sex and years of education) factors, and follow up time relative to death (months). Results with $p<0.05$ are indicated by (**) and were not adjusted for multiple comparisons, otherwise results were not significant. Abbreviations: ADNC = Alzheimer's disease neuropathological change, LATE-NC = Limbic-predominant age-related TDP-43 encephalopathy neuropathological change, MMSE = Mini-mental state examination, NPI = Neuropsychiatric inventory, M=males, F=females.

NPI score	Unadjusted model β (95% CI)		Adjusted model β (95% CI)						
	Group (vs mixed)		Group (vs mixed)		Sex (M vs F)	Age (years)	Years of education	MMSE	Time (months)
	Pure ADNC	Pure LATE-NC	Pure ADNC	Pure LATE-NC					
Delusions	0.2 (-0.7-1.0)	-0.4 (-1.8-0.9)	-0.4 (-2.3-1.4)	-0.4 (-2.8-1.9)	1.8 (0.4-3.2), p=0.013**	0.0 (-0.0-0.1)	-0.1 (-0.3-0.1)	-0.1 (-0.1- -0.0), p=0.006**	-0.0 (-0.0-0.0)
Hallucinations	0.5 (-0.3-1.2)	0.3 (-0.2-1.0)	1.0 (-0.6-2.4)	0.5 (-1.4-2.3)	0.9 (-0.2-2.1)	0.0 (-0.0-0.1)	-0.1 (-0.3-0.0)	-0.1 (-0.1- -0.0), p=0.021**	-0.0 (-0.0-0.0)
Agitation	0.4 (-0.8-1.6)	-0.2 (-2.1-1.7)	2.3 (0.7-3.8), p=0.005**	0.9 (-1.1-2.9)	-0.0 (-1.2-1.2)	0.0 (-0.0-0.1)	-0.1(-0.3-0.0)	-0.1 (-0.2- -0.0), p=0.001**	-0.0 (-0.0-0.0)
Depression	0.5 (-0.4-1.3)	0.3 (-1.1-1.6)	1.1 (-0.7-2.8)	0.7 (-1.5-2.9)	0.5 (-0.8-1.8)	0.0 (-0.1-0.1)	-0.2 (-0.4- -0.0), p=0.034**	0.0 (-0.1-0.1)	0.0 (-0.0-0.0)
Anxiety	0.4 (-0.4-1.2)	0.4 (-0.9-1.7)	0.5 (-1.3-2.2)	-0.7 (-2.9-1.5)	-0.1 (-1.4-1.3)	0.0 (-0.1-0.1)	-0.1 (-0.2-0.1)	-0.0 (-0.1-0.1)	-0.0 (-0.0-0.0)
Elation/euphoria	0.2 (-0.1-0.5)	0.1 (-0.3-0.5)	0.3 (-0.2-0.8)	0.4 (-0.3-1.0)	0.2 (-0.2-0.6)	0.0 (-0.0-0.0)	-0.0 (-0.1-0.0)	-0.0 (-0.0-0.0)	0.0 (-0.0-0.0)
Apathy	0.6 (-1.0-2.3)	-1.1 (-3.7-1.4)	1.1 (-0.7-2.8)	1.2 (-1.1-3.5)	-0.4 (-1.8-0.9)	0.0 (-0.1-0.1)	0.0 (-0.1-0.2)	-0.2 (-0.3- -0.1)	0.0 (-0.0-0.1)
Disinhibition	0.5 (-0.2-1.2)	1.0 (-0.1-2.1)	0.4 (-0.3-1.1)	1.5 (0.5-2.5), p=0.002**	0.2 (-0.4-0.8)	0.0 (-0.0-0.0)	-0.1 (-0.1-0.0)	-0.0 (-0.1—0.0), p=0.020**	0.0 (-0.0-0.0)

Irritability	0.6 (-0.4-1.6)	0.5 (-1.2-2.1)	1.4 (-0.6-3.4)	0.9 (-1.5-3.5)	0.1 (-1.4-1.6)	0.1 (-0.0-0.2)	-0.1 (-0.3-0.1)	-0.1 (-0.1- -0.0), p=0.029**	0.0 (-0.0-0.0)
Motor disturbance	0.4 (-1.0-1.8)	-1.1 (-3.3-1.2)	1.5 (0.1-2.9)	0.4 (-1.4-2.3)	0.2 (-0.9-1.3)	0.1 (-0.0-0.1)	-0.1 (-0.2-0.1)	-0.1 (-0.2- -0.1)	0.0 (-0.0-0.0)
Sleep	1.0 (-0.4-2.4)	-0.2 (-2.4-2.0)	1.6 (-0.3-3.5)	1.8 (-0.6-4.2)	0.5 (-0.9-1.9)	0.0 (-0.1-0.1)	-0.0 (-0.2-0.2)	-0.1 (-0.2- -0.0), p=0.004**	0.0 (-0.0-0.0)
Appetite	-0.4 (-1.5-0.6)	-0.3 (-2.1-1.4)	0.1 (-1.3-1.5)	1.2 (-0.7-3.0)	0.2 (-0.9-1.3)	0.0 (-0.0-0.1)	-0.1 (-0.3-0.0)	-0.0 (-0.1-0.0)	0.0 (-0.0-0.0)
